# Prediction of nickel concentration in peri-urban and urban soils using hybridized empirical bayesian kriging and support vector machine regression

**DOI:** 10.1038/s41598-022-06843-y

**Published:** 2022-02-22

**Authors:** Prince Chapman Agyeman, Ndiye Michael Kebonye, Kingsley John, Luboš Borůvka, Radim Vašát, Olufadekemi Fajemisim

**Affiliations:** 1 Department of Soil Science and Soil Protection, Faculty of Agrobiology, Food and Natural Resources, Czech University of Life Sciences Prague, 16500 Prague, Czech Republic; 2 Department of Plants Protection, Faculty of Agrobiology, Food and Natural Resources, Czech University of Life Sciences Prague, 16500 Prague, Czech Republic

**Keywords:** Ecological modelling, Urban ecology, Environmental sciences, Environmental chemistry

## Abstract

Soil pollution is a big issue caused by anthropogenic activities. The spatial distribution of potentially toxic elements (PTEs) varies in most urban and peri-urban areas. As a result, spatially predicting the PTEs content in such soil is difficult. A total number of 115 samples were obtained from Frydek Mistek in the Czech Republic. Calcium (Ca), magnesium (Mg), potassium (K), and nickel (Ni) concentrations were determined using Inductively Coupled Plasma Optical Emission Spectroscopy. The response variable was Ni, while the predictors were Ca, Mg, and K. The correlation matrix between the response variable and the predictors revealed a satisfactory correlation between the elements. The prediction results indicated that support vector machine regression (SVMR) performed well, although its estimated root mean square error (RMSE) (235.974 mg/kg) and mean absolute error (MAE) (166.946 mg/kg) were higher when compared with the other methods applied. The hybridized model of empirical bayesian kriging-multiple linear regression (EBK-MLR) performed poorly, as evidenced by a coefficient of determination value of less than 0.1. The empirical bayesian kriging-support vector machine regression (EBK-SVMR) model was the optimal model, with low RMSE (95.479 mg/kg) and MAE (77.368 mg/kg) values and a high coefficient of determination (R^2^ = 0.637). EBK-SVMR modelling technique output was visualized using a self-organizing map. The clustered neurons of the hybridized model CakMg-EBK-SVMR component plane showed a diverse colour pattern predicting the concentration of Ni in the urban and peri-urban soil. The results proved that combining EBK and SVMR is an effective technique for predicting Ni concentrations in urban and peri-urban soil.

## Introduction

Nickel (Ni) is regarded as a micronutrient for plants due to its contribution to atmospheric nitrogen (N) fixation and urea metabolism, both of which are needed for the germination of seed^1^. Apart from its contribution to seed sprouting, Ni also acts as an inhibitor for fungi and bacteria and promotes plant development. The deficiency of Ni in the soil for plants to uptake results in leaves showing chlorosis symptoms. Cowpeas and green beans, for example, require the application of Ni-based fertilizer to optimize N fixation^2^. The continuous application of Ni-based fertilizer to enrich the soil and increase the potency of the leguminous plant to fix N in the soil successively increases Ni concentration in the soil. Even though Ni serves as a micronutrient for plants, its excesses in the soil cause more harm than good. The toxicity of Ni in the soil minimizes the pH level in the soil and impedes iron uptake as an essential nutrient for plant growth^1^. According to Liu^3^, Ni has been discovered as the 17th important element required for plant development and growth. Apart from Ni playing a role in plant development and growth, humans also need it for various applications. The use of nickel in various industrial sectors is required for electroplating, the production of nickel-based alloys, and the manufacture of ignition devices and spark plugs for the automobile industries^4^. Furthermore, Ni-based alloys and plated items have been widely utilized extensively in kitchen wares, fittings for ballroom, for goods in the foods industry, electricals, wires and cables, turbines for jets, implants for surgical, textiles and building ships^5^. Enriched Ni levels in the soil (i.e. surface soil) are attributed to anthropogenic and natural sources, but primarily, Ni is of natural source than anthropogenic^4,6^. The natural sources of nickel include volcanic eruptions, vegetation, forest fires, and geological processes; however, anthropogenic sources include steel industry Ni/Cd batteries, electroplating, arc welding, diesel oil and fuel oil, and atmospheric nickel accumulation from coal combustion and waste and sludge incineration^7,8^. According to Freedman and Hutchinson^9^ and Manyiwa et al.^10^ the primary source of topsoil pollution in the immediate vicinity and adjacent environments is principally caused by Ni-Cu based smelter and mines. The topsoil around the Ni–Cu refinery in Sudbury, Canada, had the highest levels of Ni pollution, up to 26,000 mg/kg^11^. In contrast, pollution from Russia's nickel production has culminated in higher Ni concentrations in Norwegian soils^11^. According to Alms et al.^12^ the quantities of HNO_3_-extractable Ni in top cultivated fields in this area (Russia nickel production) ranged from 6.25 to 136.88 mg/kg with a corresponding of mean 30.43 mg/kg, and a baseline concentration of 25 mg/kg. The application of phosphate fertilizer to agricultural soil in urban or peri-urban soils to successive crop seasons injects or pollutes the soil, according to kabata^11^. The potential impact of nickel on humans potentially cause cancer via mutagenesis, chromosomal damage, Z-DNA creation, obstruction of DNA excision repair, or epigenetic processes^13^. In animal experiments, nickel has been found to have the potential to cause a variety of tumours, which can be exacerbated by carcinogenic nickel complexes^14^.

Soil pollution assessment is prevalent in the recent era because of the wide range of health-related issues that arise from soil–plant relationships, soil and soil organism relationships, ecological degradation and environmental impact assessment related issues. Hitherto, spatial prediction of potentially toxic elements (PTEs) such as Ni in the soil using conventional means has been laborious and time-consuming. The advent of digital soil mapping (DSM) and its success chalked^15^ in this present time has improved predictive soil mapping (PSM) tremendously. Predictive soil mapping, or DSM, according to Minasny and McBratney^16^ has proven to be a prominent soil science subdiscipline. Lagacherie and McBratney, 2006 define DSM as "the *creation and population of spatial soil information systems by using field and laboratory observational methods coupled with spatial and non-spatial soil inference systems"*. McBratney et al.^17^ outlined that DSM or PSM in contemporary time is the utmost effective technique to foretell or map the spatial distribution of PTEs, types of soil and soil properties. Geostatistics and machine learning algorithms (MLA) are DSM modeling techniques that use significant and minimal data to create a digitized map with the assistance of a computer.

Deutsch^18^ and Olea^19^ defines geostatistics “as *a collection of numerical techniques that deal with the characterization of spatial attributes, employing primarily random models like how time series analysis characterizes temporal data*.” Mainly, geostatistics involves the assessment of variograms, which allows quantifying and defining the dependency of spatial values from every sort of dataset^20^. Gumiaux et al.^20^ further illustrated that the assessment of variogram in geostatistics is based on the three principles, including (a) to compute the data correlation scale, (b) to identify and calculate the anisotropies in the disparity of the dataset and (c) estimate the area effects in addition to intrinsic errors that takes in accounts measured data that is segregated from local effects. On the basis of these concepts, numerous interpolation techniques including as universal kriging, cokriging, ordinary kriging, empirical bayesian kriging, simple kriging, and other well-known interpolation techniques are employed within geostatistics to map or predict PTEs, soil characteristics, and soil types.

Machine learning algorithms (MLAs) are a relatively new technique that employs larger nonlinear data classes propelled by algorithms primarily used for data mining, identifying data patterns, and repeatedly applied to classification and regression tasks in scientific fields such as soil science^21^. Substantial research papers have relied on MLA models to predict PTEs in soil, such as Tan et al.^22^ (random forest for heavy metal estimation in agricultural soil), Sakizadeh et al.^23^ (application of support vector machine and artificial neural network to model soil pollution). Furthermore, Vega et al.^24^ (CART for modelling heavy metal retention and sorption in soil) Sun et al.^25^ (application of cubist is the distribution of Cd in the soil) and other algorithms like *k*-nearest neighbours, generalized boosted regression and boosted regression tree also applied MLA to predict PTEs in the soil.

The application of DSM algorithms in prediction or mapping comes with several challenges. Many authors have argued the superiority of MLA to geostatistics and contrariwise. Even though one is superior to the other, the combination of the two has increased the accuracy level in mapping or prediction in DSM^15^. Woodcock and Gopal^26^ Finke^27^; Pontius and Cheuk^28^ and Grunwald^29^ have commented on the imperfection and some errors that exist in predictive soil mapping. Soil scientists have tried a variety of techniques to optimize the effectiveness, precision, and predictability of DSM mapping and prediction. The incorporation of uncertainty and validation is one of the many different facets that have been integrated into DSM to optimize effectiveness and decrease imperfection. Nevertheless, Agyeman et al.^15^ outlined that the act of validation and the uncertainty that come with the creation of map and prediction should be validated independently to enhance map quality. The limitations in DSM are due to geographically dispersed soil qualities, which involve a component of uncertainty^30^; nevertheless, the lack of certainty in DSM may stem from multiple error sources, namely covariate error, model error, positional error and analytical error^31^. The modelling inaccuracy triggered during MLA and geostatistical relates to a lack of comprehension, culminating in an oversimplification of genuine processes^32^. Regardless of the nature of modelling, the inaccuracy can be attributed to modelling parameters, mathematical model predictions, or interpolations^33^. Recently, there has been a new DSM trend that fosters the combination of geostatistics and MLA in mapping and prediction. Several soil scientists and authors such as Sergeev et al.^34^; Subbotina et al.^35^; Tarasov et al.^36^and Tarasov et al.^37^ have harnessed accurate qualities in geostatistics and machine learning to generate hybrid models that increase the efficiency and quality of the prediction as well as mapping. Some of these hybridizations or combined algorithmic models are artificial neural network-kriging (ANN-RK), multi-layer perceptron residual kriging (MLP-RK), generalized regression neural network residual kriging (GR-NNRK)^36^, artificial neural network-kriging- multilayer perceptron (ANN-K- MLP)^37^ and cokriging and gaussian process regression^38^.

According to Sergeev et al., combining various modelling techniques has the potential to eliminate flaws and increase the efficiency of the hybrid model produced over the single models from which it was developed. Against this backdrop, this new paper deems it necessary to apply a combined algorithm from geostatistic and MLA to create the best-hybridized model to predict the enrichment of Ni in the urban and peri-urban area. This research will lean on empirical Bayesian kriging (EBK) as the base model and hybridize it with a support vector machine (SVM) as well as multiple linear regression (MLR) model. The hybridization of EBK with any MLA is uncharted. The plurality of hybrid models seen is a combination of ordinary, residual, regression kriging and MLA. EBK is a geostatistical interpolation approach that utilizes a spatial stochastic process that is localized as a non-stationary/stationary random field with a defined localize parameter on the field that allows for space variation^39^. EBK has been applied in a variety of studies, including the analysis of the distribution of organic carbon in agrogray soils^40^, soil contamination assessment^41^ and mapping soil properties^42^.

On the other hand, a self-organising map (SeOM) is a learning algorithm that has been applied in various articles such as Li et al.^43^, Wang et al.^44^, Hossain Bhuiyan et al.^45^, and Kebonye et al.^46^ to determine the spatial attributes and grouping of elements. Wang et al.^44^ outlined that SeOM is a vigorous learning technique known for its capacity in grouping and imagining that is allowed to deal with nonlinear problems. SeOM, unlike other pattern recognition techniques such as principal component analysis, fuzzy clustering, hierarchical clustering and multiple criteria decision making, performs better in an organization and recognising the pattern of PTEs. According to Wang et al.^44^, SeOM can spatially group the distribution of related neurons and provide high-resolution data visualization. SeOM will visualize Ni prediction data for the best model developed to characterize the results for straightforward interpretation.

This paper intends to generate a robust mapping model with optimal accuracy that predicts Ni content in urban and peri-urban soil. We hypothesized that the dependability of the hybridized model primarily relies on the influence of the other model attached to the base model. We acknowledge the challenges in DSM, and while these challenges are being addressed on multiple fronts, the combination of geostatistics and MLA model progression appears to be gradual; therefore, we will attempt to answer the research question that may generate a hybrid model. Nevertheless, how accurate is the model in predicting the targeted element? Furthermore, what is the efficiency assessment level based on validation and accuracy assessment? Therefore, the specific objectives of this research are (a) to create a combined hybrid model using EBK as the base model against SVMR or MLR, (b) compare the models generated (c) propose the best hybrid model to predict the concentration of Ni in urban or peri-urban soil and (d) to apply SeOM to create high-resolution spatial variability maps of Nickel.

## Materials and methods

### Study area

**Figure 1:**
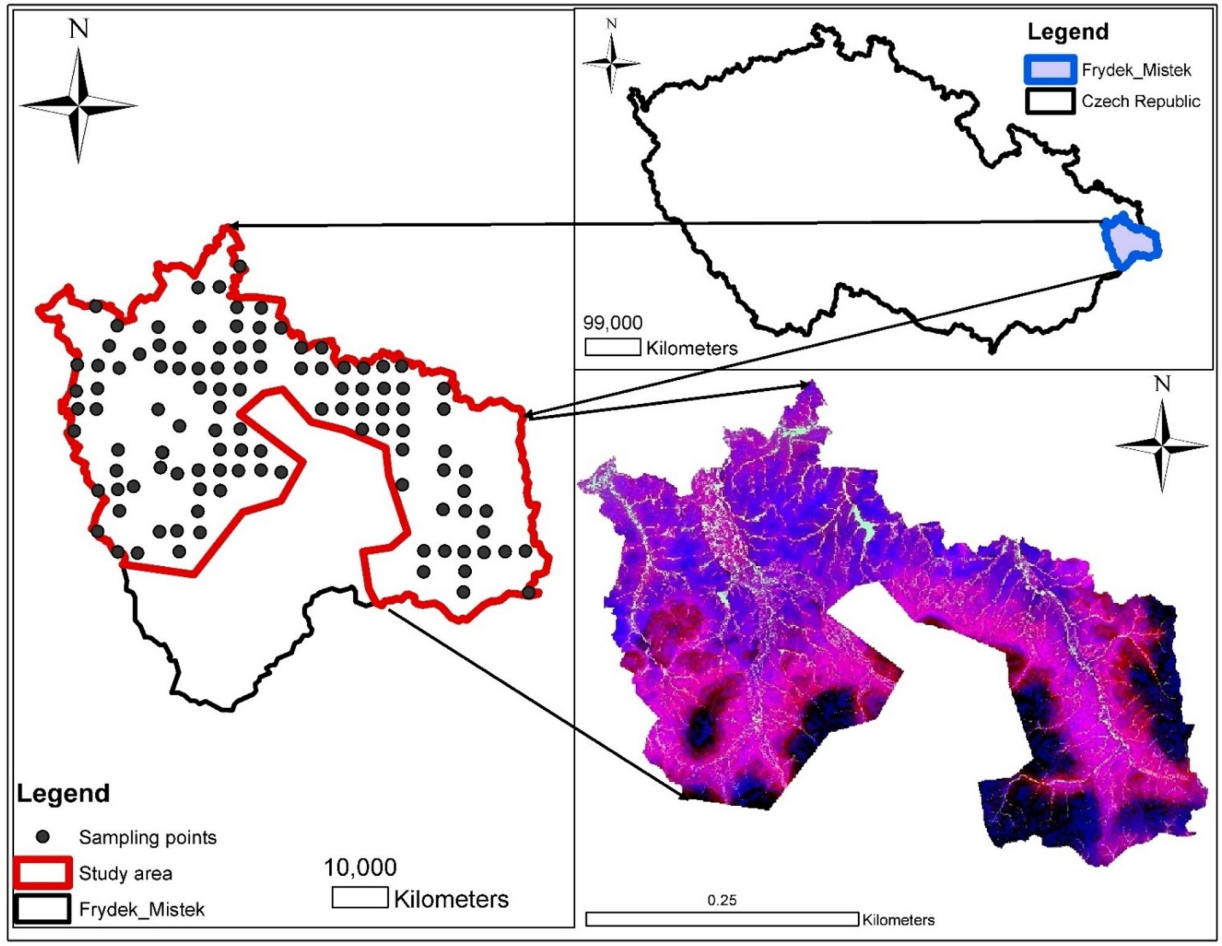
Study area map [The study area maps was created with ArcGIS Desktop (ESRI, Inc, Version 10.7, URL: https://desktop.arcgis.com).]

The research is being conducted in the Czech Republic, specifically in the Frydek Mistek district of the Moravian-Silesian Region (see Fig. 1). The study area's geography is a very rugged landscape that is mostly part of the Moravian-Silesian Beskydy region, which is part of the outer Carpathian Mountain range. The study area falls within latitude 49° 41′ 0′ North and longitude 18° 20′ 0′ East at an altitude varying between 225 and 327 m above sea level; however, the Koppen classification system of the area's climatic situation is rated as Cfb = temperate oceanic climate with a high amount of rainfall even in dry months. Temperatures vary slightly between − 5 °C and 24 °C throughout the year and are seldom below − 14 °C or above 30 °C, whereas average annual precipitation is between 685 and 752 mm^47^. The district's area survey is projected to be 1208 km^2^, with 39.38% of the land under cultivation and 49.36% under forest cover. The area used for this study, on the other hand, is approximately 889.8 km^2^. In and around the Ostrava neighbourhood, the steel industry and metal works are active. Metal works, steel industry that uses Ni for stainless steel (e.g., resisting corrosion from the atmosphere) and alloy steel (nickel can increase the strength of the alloy while maintaining its good plasticity and toughness), and intensive agriculture such as phosphate fertilizer application and livestock production are potential sources of Ni in the study area (e.g., Ni supplement in sheep lamb to increase growth rate in lambs and cattle fed low). Other industrial uses of Ni in the research area include its usage in electroplating, which consists of the electroplated nickel and electroless nickel processes. The soil properties are easily differentiated from the soil's colour, structure, and carbonate content. The soil's texture is medium to fine, and it is derived from parent materials. They are either colluvial, alluvial, or aeolian in nature. Some soil areas show mottles in the top and subsoil, which are usually accompanied by concrete and bleaching. However, cambisols and stagnosols are the most common soil types in the area^48^. With elevations ranging from 455.1 to 493.5 m, cambisols is predominate in the Czech Republic^49^.

### Soil sampling and analysis

Topsoil samples totaling 115 were obtained from urban and peri-urban soil in the Frydek Mistek district. The sample pattern used was the regular grid, and the soil sample intervals were 2 × 2 km using a handheld GPS device (Leica Zeno 5 GPS) at a depth of 0 to 20 cm for topsoil. The samples were wrapped in Ziploc bags, labelled appropriately, and transported to the laboratory. The samples were air-dried to produce a pulverized sample, crushed by a mechanical system (Fritsch disk mill), and sieved (sieve size 2 mm). A gram of the dried, homogenized, and sieved soil sample was placed in a Teflon bottle that was clearly labelled. In each Teflon container, 7 ml of 35% HCl and 3 ml of 65% HNO_3_ were dispensed (using automatic dispensers—one for each acid), and the cap was gently closed to allow the sample to remain overnight for reactions (aqua regia procedure). The supernatant was put on a hot metal plate (temperature: 100 Watt and 160 °C) for 2 h to promote the digestion process of the sample before being allowed to cool. The supernatant was transferred to a 50 ml volumetric flask and diluted to 50 ml with deionized water. After that, the diluted supernatant was filtered into 50 ml PVC tubes with deionized water. In addition, 1 ml of the diluted solution was diluted with 9 ml of deionized water and filtered into a 12 ml test tube prepared for PTE pseudo-concentration in this study. The concentration of PTEs (As, Cd, Cr, Cu, Mn, Ni, Pb, Zn, Ca, Mg, K) was determined by ICP-OES (inductively coupled plasma optical emission spectrometry) (Thermo Fisher Scientific, USA) following standard methods and protocols. The quality assurance and control (QA/QC) procedure was ensured (SRM NIST 2711a Montana II soil). PTEs having detection limits of less than half were excluded from this study. The PTE used in this study had a detection limit of 0.0004. (Ni). Furthermore, the quality control and quality assurance process for each analysis was ensured by analyzing the reference standards. To ensure that the error was minimized, a double analysis was performed.

### Empirical Bayesian kriging

Empirical Bayesian kriging (EBK) is one of the numerous geostatistical interpolation techniques used in modelling in diverse fields such as soil science. Unlike the other kriging interpolation techniques, EBK varies from conventional kriging methods by considering the error of the semivariogram model estimation^50^. In EBK interpolation, several semivariogram models, are calculated during the interpolation instead of a unitary semivariogram. The interpolation technique makes way for uncertainties associated with this plotting semivariogram and programming the highly complex parts of composing a sufficient kriging approach^40^. The interpolation process of EBK follows three criteria as proposed by Krivoruchko^50^, (a) the model estimate semivariogram from the input dataset (b) based on the generated semivariogram a new predicted is value against each inputted dataset location and (c) finally a model is computed from the simulated dataset. The bayesian equation rule is given as posterior

1 $$Prob\left(A,B\right)= Prob\left(\frac{A}{B}\right)Prob\left(B\right)= Prob\left(B,A\right)=P\left(\frac{B}{A}\right)P\left(A\right)$$

where the $Prob\left(A\right)$ represents the prior, $Prob\left(B\right)$ marginal probability in the most instances there they are ignored, $Prob (B,A)$ the posterior. The semivariogram calculation is based on the Bayes rule, which exhibits the proclivity that the observed dataset can be created from the semivariogram. The semivariogram's value is subsequently determined employing Bayes' rule, which illustrates how probable the observed dataset could be created from the semivariogram.

### Support vector machine regression

Support vector machine is a machine learning algorithm that generates an optimal disengaging hyperplane to differentiate identical but not linearly independent categories. Vapnik^51^, created the algorithm for intent classification, but it has recently been used to solve regression-oriented problems. According to Li et al.^52^, SVM is one of the best classifier techniques and has been used in various fields. The regression component of SVM is used in this analysis (support vector machine regression-SVMR). Cherkassky and Mulier^53^, pioneered SVMR as a regression based on the kernel, and its computation was performed using a linear regression model with a multinational space function. John et al.^54^ reported that the SVMR modelling employs a hyperplane linear regression, which creates a nonlinear relationship and allows for the space function. According to Vohland et al.^55^ epsilon (ε)-SVMR uses a trained dataset to obtain a represented model as an epsilon -insensitive function applied to map data independently with the optimum epsilon-deviation from dependent data training. The preset distance error within is ignored from the actual value, and if the error is larger than the epsilon (ε), the soil property compensates for it. The model also reduces the intricacy of training data to a broader subset of support vectors. The equation as proposed by Vapnik^51^ is given as.

2 $$y\left(x\right)= {\sum }_{k=1}^{N}{\alpha }_{k} \, K\left({x}_{,}{ x}_{k}\right)+ {b}_{,}$$

In which the b represents the scalar threshold, $K\left({x}_{,}{ x}_{k}\right)$ representing the kernel function, $\alpha$ denoting the Lagrange multiplier, N symbolizing the number dataset, ${x}_{k}$ representing the data input, and $y$ is the data output. One of the critical kernels used is the SVMR operation with is the gaussian radial basis function (RBF). The RBF kernel was applied to ascertain the optimum SVMR model essential to procure the most nuanced penalty set factors C and the kernel parameters gamma (γ) for the PTE training data. First, we assessed the set of training and then tested the validation set's model performance. The turning parameter used was sigma and the method value was svmRadial.

### Multiple linear regression

The multiple Linear Regression Model (MLR) is a regression model that embodies the relationship between a response variable and numerous predictor variables by employing linearly incorporated parameters that are computed using the least-squares method. In MLR, the least square model is a prediction function that is directed toward a soil property following the selection of an explanatory variable. It was necessary to use the response in building a linear relationship using the explanatory variable. The PTE was used as the response variable which was used to establish the linear relationship utilizing the explanatory variable. The MLR equation is given as

3 $$y=a+ \sum_{i-1}^{n}{b}_{1 } \, \mathrm{\rm X}\, {x}_{i} \pm {\varepsilon }_{i}$$

In which the y represents the response variable, $a$ denotes the intercept, n signifies the number of predictors, ${b}_{1}$ denotes the partial regression of coefficient, ${x}_{i}$ implies the predictors or the explanatory variables and the ${\varepsilon }_{i}$ signifies the error in the model, which is also called residual.

The model was utilized in RStudio.

### Hybrid modelling

**Figure 2:**
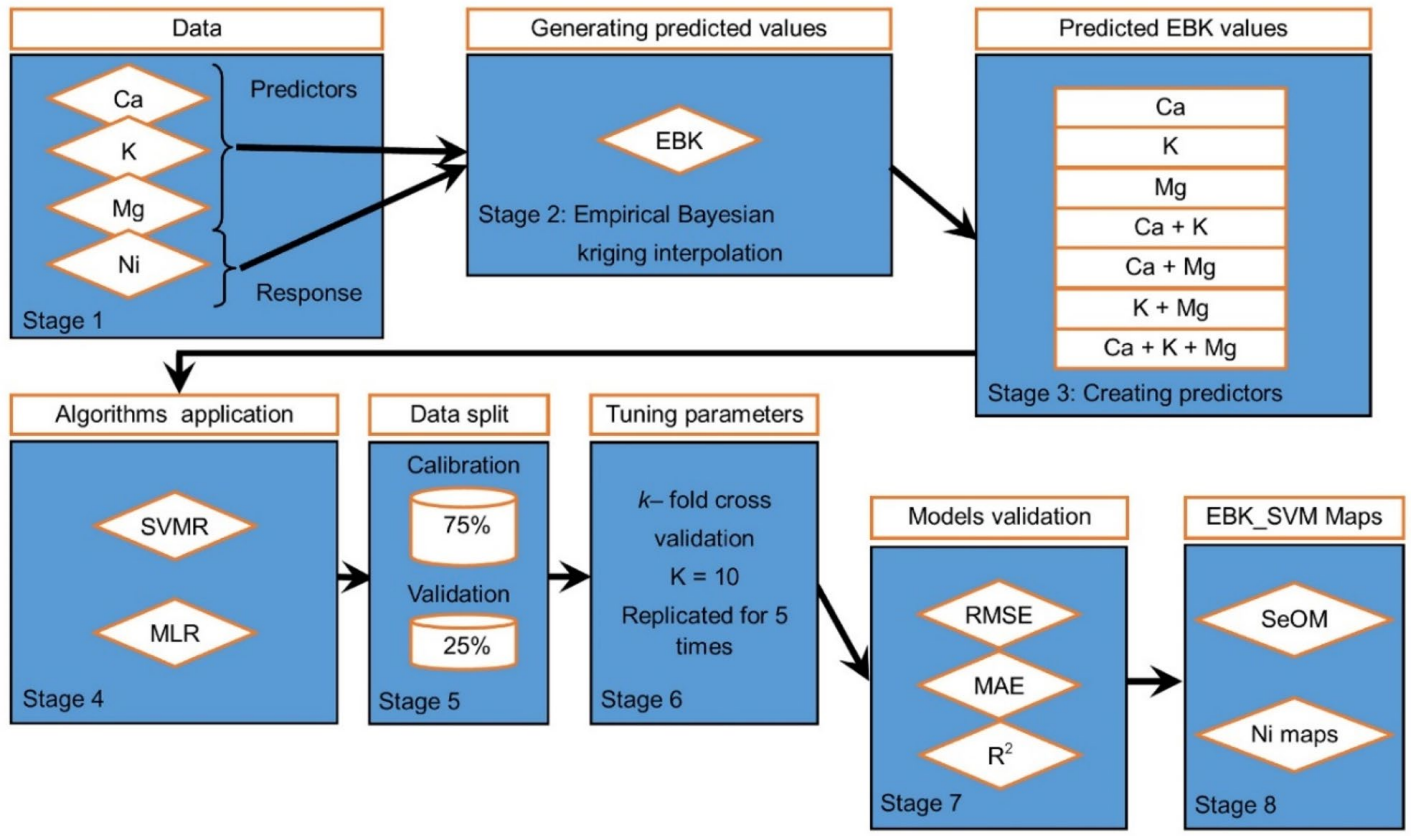
Flowchart of the study.

The hybrid models were obtained by sandwiching the EBK as the base model with SVMR and MLR. This was done by extracting predicted values from the EBK interpolation. The predicted values obtained from interpolated Ca, K and Mg were passed through a combination process to obtain new variables such as CaK, CaMg and KMg. The elements Ca, K and Mg were then combined to obtain the fourth variable, CaKMg. Overall, the variables obtained were Ca, K, Mg, CaK, CaMg, KMg and CaKMg. These variables became our predictors that will aid in predicting Nickel concentration in urban and peri-urban soil. The predictors were subjected to an SVMR algorithm to obtain a hybrid model Empirical bayesian kriging—Support vector machine (EBK_SVM). Similarly, the variables were piped through MLR algorithm likewise to obtain a hybrid model Empirical bayesian kriging -multiple linear regression (EBK_MLR). Generally, the variables Ca, K, Mg, CaK, CaMg, KMg and CaKMg were used as covariates which served as predictors in predicting the Ni content in urban and peri-urban soil. The most acceptable model (EBK_SVM or EBK_MLR) obtained will then be visualized using the self-organizing map. The workflow of the study is presented in Fig. 2.

### Self-organizing maps (SeOM)

Using SeOM has become a popular tool in a variety of sectors for the organization, appraisal, and prediction of data in the financial sector, medical sector, industrial sector, statistics, soil science, and so on. SeOM was created using an artificial neural network for organization, evaluation, and prediction, as well as unsupervised learning approaches. In this study, SeOM was used to visualize the concentration of Ni based on the finest model used to predict Ni in urban and peri-urban soil. The data treated in the SeOM assessment serves as an n input dimensional vector variable^43,56^. Melssen et al.^57^ delineated that an input vector is connected to an output vector with a single weight vector by a single input layer into a neural network. The output generated from SeOM comes out as a two-dimensional map made up of diverse neurons or nodes knitted together into either a hexagonal, circular or square topological plot based on their proximity^43^. Map sizes were compared based on metrics, quantization error (QE) and topographic error (TE), and a SeOM model with 0.086 and 0.904 respectively was chosen, which was a 55-map unit (5 × 11). The neuron structure was determined based on empirical equation node number given as

$$m=5\times \sqrt{n}$$

In which the m denotes the quantity of SeOM map neurons, n represents the input data quantity.

### Data partitioning

The number of data used in this study is 115 samples. A random method was employed to dissect the data into test data (25% for validation) and a training dataset (75% for calibration). The training dataset was used to produce the regression models (calibration), and the test dataset was used to validate generalization capabilities^58^. This was done to evaluate the appropriateness of the diverse models that are being used to predict nickel content in the soil. All the models used were subjected to a tenfold cross-validation process that was replicated five times. The variables generated from EBK interpolation were used as the predictors or explanatory variables to predict the targeted variable (PTEs). The modelling was processed in RStudio, and the packages utilized were library (Kohonen), library(caret), library(modelr), library ("e1071"), library("plyr"), library("caTools"), library("prospectr"), and library ("Metrics").

### Model performance metrics

A variety of validation parameters were used to determine the optimal model suitable for the prediction of nickel concentration in the soil and evaluate the accuracy of the model and its validation. The hybridized models were assessed using mean absolute error (MAE), root means square error (RMSE), and R square, or coefficient determination (R^2^). R^2^ defines the variance of the proportion in the answer and is represented by the regression model. The RMSE and the magnitude of the variance within the independent measurement describe the model prediction power, while MAE determines the actual quantitative value. The R^2^ value must be high to evaluate the best-hybridized model using the validation parameters, and the closer the value is to 1, the higher the accuracy. According to Li et al.^59^an R^2^ criteria value of 0.75 or greater is considered a good prediction; from 0.5 to 0.75 is acceptable model performance and below 0.5 is unacceptable model performance. A lower obtained value is sufficient and considered best for selecting a model using the RMSE and MAE validation criteria evaluation methods. The following equation describes the validation methods.

Mean absolute error

$$MAE= \frac{1}{n}\sum_{i=1}^{n}{Y}_{i}- {\widehat{Y}}_{i}$$

R square

$${R}^{2} =1-\frac{\sum_{i=1}^{n}({\widehat{Y}}_{i} - {Y}_{i} {)}^{2}}{\sum_{i=1}^{n}({Y}_{i - }{\widehat{Y}}_{i}{)}^{2}} $$

Root mean square error

$$RMSE\left(mg/kg\right)=\sqrt{\frac{1}{n}} \sum_{i=1}^{n}( {\widehat{\mathrm{Y}}}_{\mathrm{i}}- {Y}_{i}{)}^{2}$$

whereby n represents the size of the observations ${Y}_{i}$ represents the measured response and the ${\widehat{Y}}_{i}$ also stated as the predicted response values, accordingly, for the ith observation term.

## Results and discussion

### Statistical description

The statistical description of the predictors and the response variables are shown in Table 1, displaying the mean, standard deviation (SD), coefficient of variability (CV), minimum value, maximum value, kurtosis and skewness. The elements minimum and maximum values descend in this Mg < Ca < K < Ni and Ca < Mg < K < Ni order, respectively. The concentration of the response variable (Ni) sampled from the study area ranged from 4.86 to 42.39 mg/kg. Comparing Ni with the world average value (29 mg/kg) and the European average value (37 mg/kg) indicates that the overall computed geometric mean of the study area is under tolerable limits. Nevertheless, comparing the mean concentration of nickel (Ni) in this current study to the agricultural soils in Sweden, as indicated by Kabata-Pendias^11^ exhibits that the current mean concentration of Ni is higher. Similarly, the mean concentration in the current study (Ni 16.15 mg/kg) of the urban and peri-urban soil in Frydek Mistek is higher than the permissible limit for Ni in urban soil in Poland as reported by Różański et al.^60^ (10.2 mg/kg). Furthermore, the mean Ni concentration in Tuscan urban soil recorded by Bretzel and Calderisi^61^ (1.78 mg/kg) is very low compared to the current study. Jim^62^ also identified a low Ni concentration in Hong Kong urban soil (12.34 mg/kg), lower than the current Ni concentration of this study. Birke et al.^63^ reported a Ni mean concentration of 17.6 mg/kg in an old mining and urban industrial area in Sachsen-Anhalt, Germany, which is 1.45 mg/kg higher than the Ni (16.15 mg/kg) mean concentration in the current study. The concentration of Ni in some parts of the study area's urban and peri-urban soil that exceeds the allowable limit might be attributed largely to steel industries and metal works. This is inline with Khodadoust et al.^64^ studies that steel industries and metal works are major sources of nickel pollution in the soil. However, the predictor variables also ranged from 538.70 mg/kg to 69,161.80 mg/kg for Ca, 497.51 mg/kg to 3535.68 mg/kg for K and 685.68 mg/kg to 5970.05 mg/kg for Mg. Jakovljevic et al.^65^ investigated the total content of Mg and K in central Serbian soil. They found that the total concentration (410 mg/kg and 400 mg/kg, respectively) was lower than the Mg and K concentration of the current study. Indistinguishably, in eastern Poland, Orzechowski and Smolczynski^66^ assessed the total content of Ca, Mg and K, and the results suggested that the mean concentration Ca (1100 mg/kg), Mg (590 mg/kg) and K (810 mg/kg) in the topsoil were lower than the individual elements in this present study. A recent study conducted by Pongrac et al.^67^ revealed that Ca total content analyzed in 3 different soil in Scotland Uk (Mylnefield soil, Balruddery soil and Hartwood soil) suggested the Ca content of the present study is higher.

**Table 1:** Statistical description of predictors and response.

	Ca	K	Mg	Ni
	mg/kg			
	Predictors			Response
Mean	3624.83	1289.75	1981.91	16.15
Standard deviation	7969.72	446.87	666.69	6.78
Coefficient of variability	219.86	34.65	33.64	41.97
Minimum value	538.70	497.51	685.68	4.86
Maximum value	69,161.80	3535.68	5970.05	42.39
Kurtosis	54.16	4.82	11.67	2.49
Skewness	7.24	1.54	2.48	1.63

The dataset distribution of the elements exhibited different skewness due to the differences in the measured concentration of the elements sampled. The skewness and the kurtosis of the elements ranged from 1.53 to 7.24 and 2.49 to 54.16 correspondingly. All the computed skewness and kurtosis levels of the elements were above + 1, and it thus indicates that the data distribution is irregular skewed in the right direction and leptokurtic. The estimated CV of the elements also suggested that K, Mg and Ni showed a moderate variability, whereas Ca had extremely high variability. The CV of K, Ni and Mg explained that they are homogeneously distributed. Moreover, Ca distribution is non-homogeneous, and an external source might influence its level of enrichment.

### Correlation between response and predictor variable

**Figure 3:**
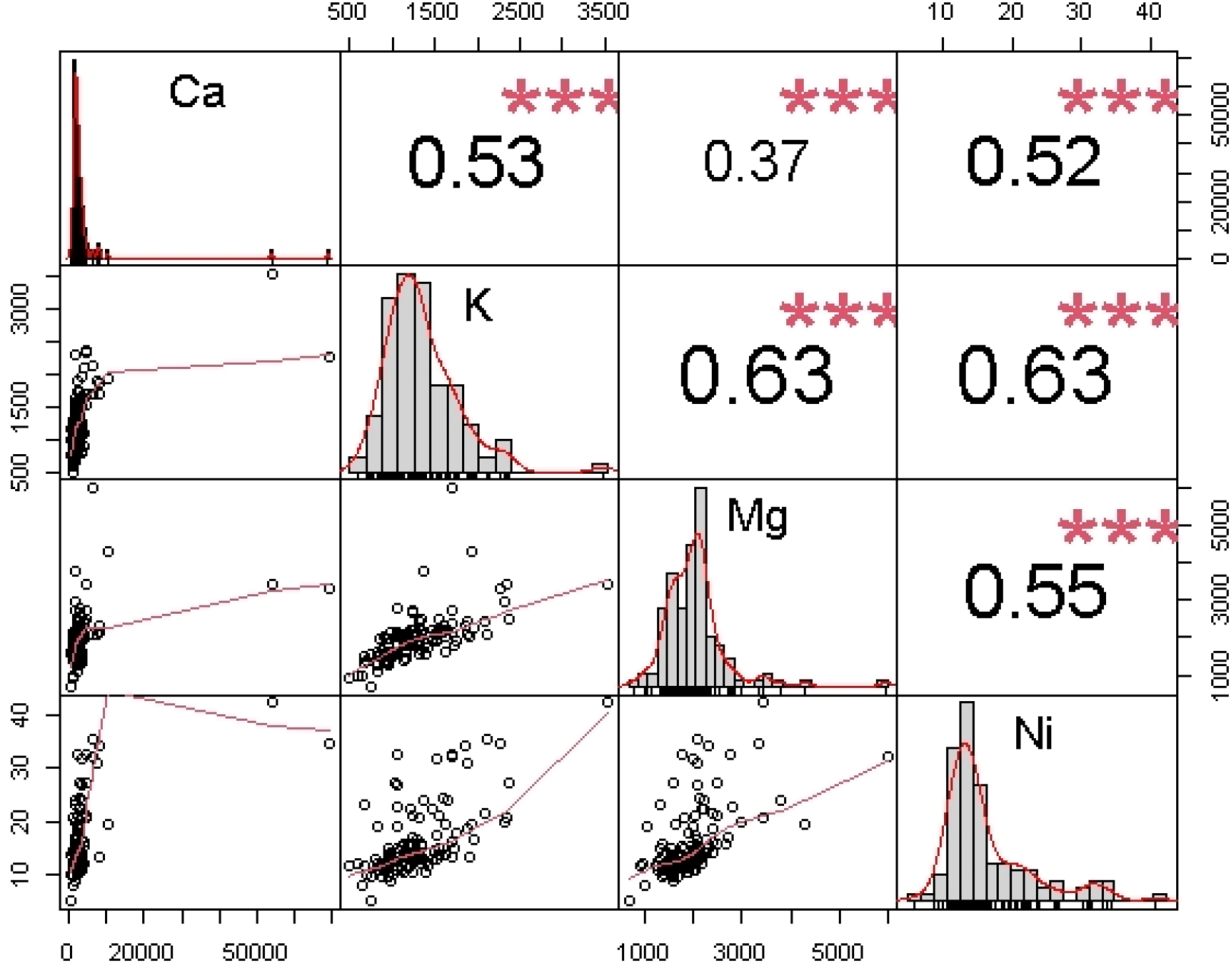
Correlation matrix of the elements showing the relationship between predictors and response (Note: The plot includes scatter plots between the element, and the significance levels is based on p < 0,001).

The correlation of the predictors against the response element suggested a satisfactory correlation among the elements (see Fig. 3). The correlation suggested that CaK showed a moderate correlation with r value = 0.53 and CaNi similarly displayed moderate correlation. Even though Ca and K showed moderate nexus, among each other but researchers such as Kingston et al.^68^ and Santo^69^ have suggested that their content in the soil is inversely proportional. However, Ca and Mg are antagonistic to K, but CaK correlated very well. This might be due to applying fertilizer such as potassium carbonate that is 56% richer in potassium. Potassium correlated moderately with magnesium (KMg r = 0.63). In the fertilizer industry, these two elements have a history of strong relationships due to applying potassium magnesium sulfate, potassium magnesium nitrate and muriate of potash to the soil to enhance its deficiency level. Nickel correlated moderately with Ca, K and Mg with r values = 0.52, 0.63 and 0.55, respectively. The relationships involving calcium, magnesium, and PTE such as nickel are complicated, but notwithstanding, magnesium inhibits calcium absorption, calcium decreases the effects of excess magnesium, and both magnesium and calcium reduce the toxicity effects of the nickel in the soil.

### Spatial distribution of the elements

**Figure 4:**
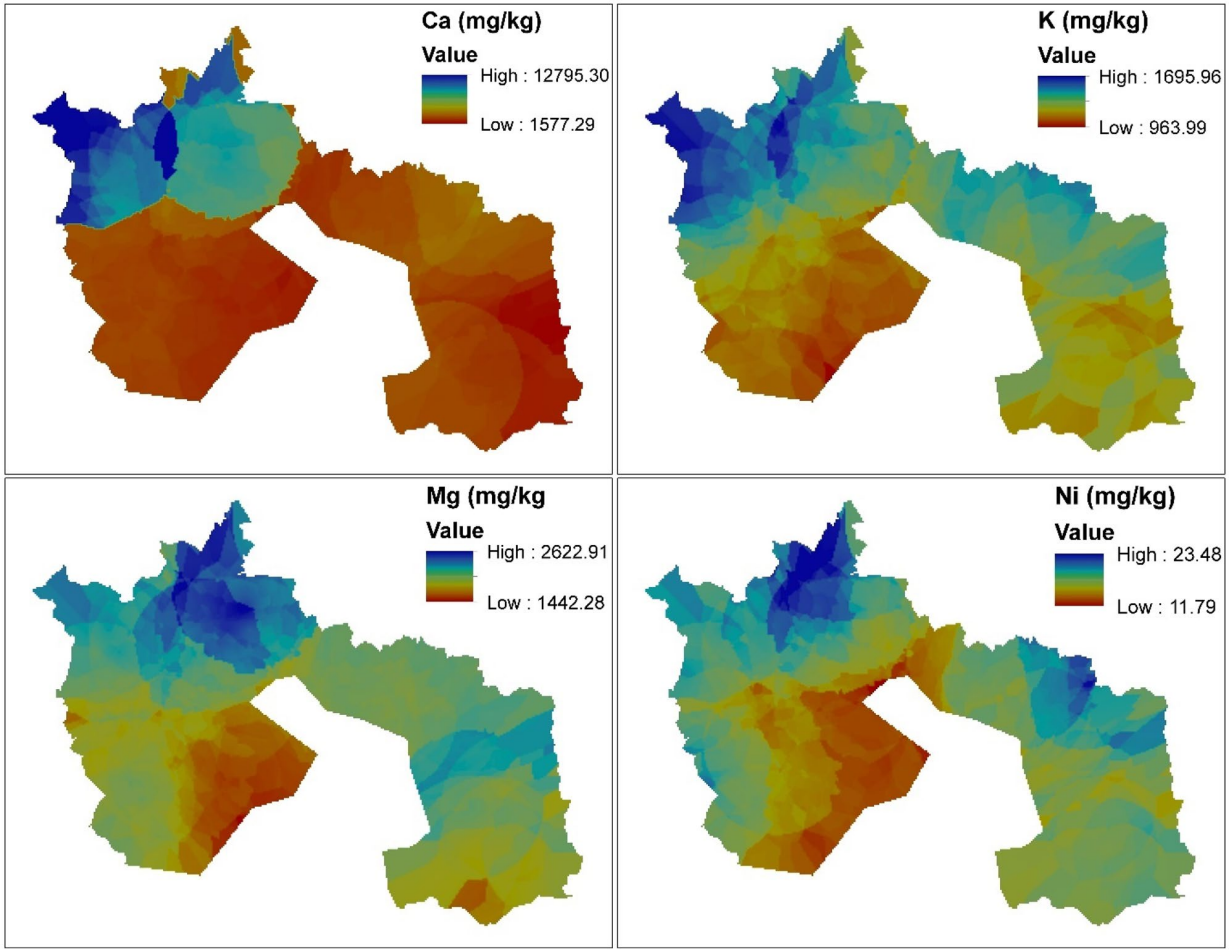
Spatial distribution of the elements [The spatial distribution maps was created with ArcGIS Desktop (ESRI, Inc, Version 10.7, URL: https://desktop.arcgis.com).]

Figure 4 illustrates the spatial distribution of the elements. According to Burgos et al.^70^ applications of spatial distribution is a technique used to quantify and highlight hot spots of polluted areas. The enrichment level of Ca in Fig. 4 can be seen in the northwestern part of the spatial distribution map. The map shows moderate to high hotspots of Ca enrichment. Calcium enrichment in the northwestern part of the map might be due to the application of quicklime (Calcium oxide) to reduce soil acidity and its application in steel plants as basic oxygen in steel making process. On the other hand, other farmers prefer to use calcium hydroxide in acidic soil to neutralize the pH level, which also surges the calcium content of the soil^71^. Potassium exhibited hot spots in the northwestern part of the map and the eastern part as well. The Northwestern part is the predominantly agrarian community, and a moderate to high pattern of K might be due to the application of NPK and muriate of potash. This is coherent with other studies such as Madaras and Lipavský^72^, Madaras et al.^73^, Pulkrabová et al.^74^, Asare et al.^75^ who observed using muriate of potash and NPK for soil stabilization and treatment resulted in high K content in the soil. Potassium enrichment in the northwestern part of the spatial distribution map might be due to the usages of potassium-based fertilizers such as potassium chloride, potassium sulphate, potassium nitrate, sylvinite, and kainit to increase the k content of deficient soil. Zádorová et al.^76^ and Tlustoš et al.^77^ outlined that the application of potassium-based fertilizer increases the potassium level in the soil and, by a long effect will significantly upsurge soil nutrient content, especially K. Magnesium showed a hot spot in the northwestern part of the map and relatively moderate hotspot in the southeastern part of the map. Colloid fixation in soil depletes the concentration of magnesium in the soil. Its deficiency in the soil causes plants to portray interveinal chlorosis of yellowish colouration. Magnesium-based fertilizers, such as potassium magnesium sulphate, magnesium sulphate and Kieserite, treat deficiency syndrome (purple, red or brown colouration of plants indicating lack magnesium) in soils with normal pH ranges^6^. The accumulation of Nickel on the surface of the urban and peri-urban soil might be due to anthropogenic activities such as agriculture and Ni importance in stainless steel production^78^.

The results of the model performance metrics of the elements used in this study are presented in Table 2. The RMSE and MAE for Ni, on the other hand, were both closer to zero (0.86 RMSE, −0.08 MAE). The RMSE and MAE values for K, on the other hand, were both acceptable. The RMSE and MAE results for calcium and magnesium were greater. Because of the dataset's dissimilarity, the Ca and K MAE and RMSE results are greater. The results of this study's RMSE and MAE for predicting Ni using EBK were found to be better than those of John et al.^54^ for predicting S concentration in soil using cokriging using the same collected data. The EBK output of our study is related to those of Fabijaczyk et al.^41^, Yan et al.^79^, Beguin et al.^80^, Adhikary et al.^81^, and John et al.^82^, especially K and Ni.

**Table 2:** Showing the EBK model performance.

	RMSE	MAE
	mg/kg	
Ca	2492.35	2072.23
Mg	142.71	− 13.31
K	9.46	− 0.88
Ni	0.86	− 0.08

### Performance of models

**Figure 5:**
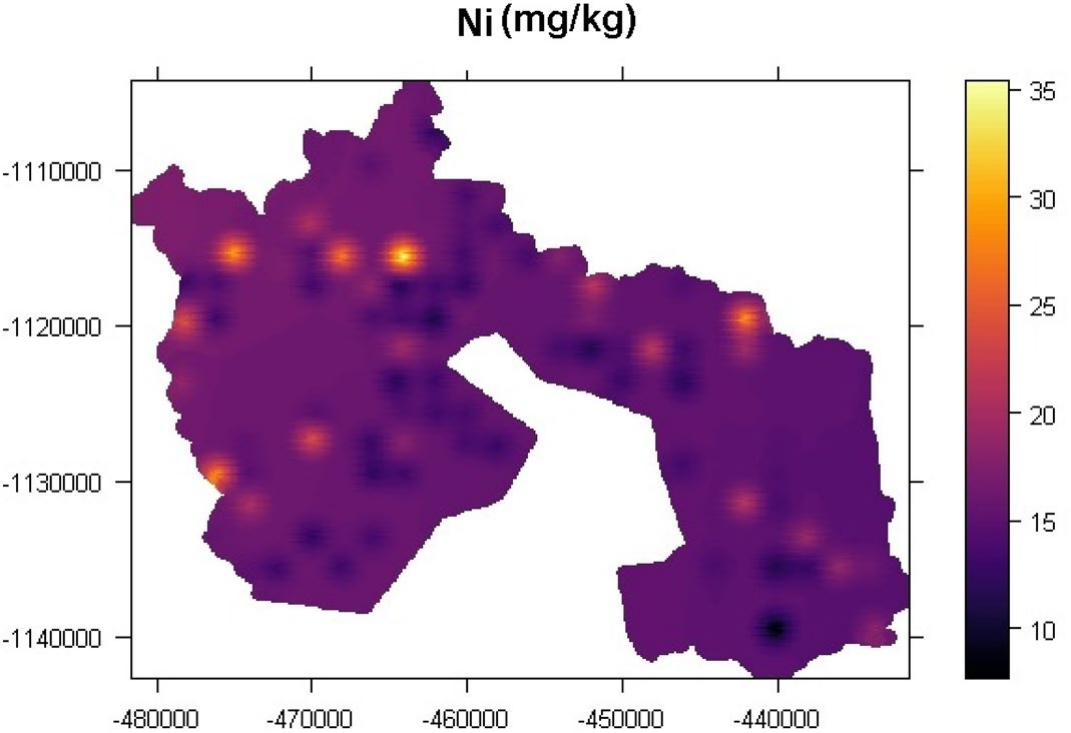
Represent the final predicted map using the hybridized model EBK _SVMR and using Ca_Mg_K as a predictor. [The spatial distribution map was created with RStudio (Version 1.4.1717: https://www.rstudio.com/).]

The performance of individual approaches for predicting Ni content in urban and peri-urban soil was assessed using the models' performance (Table 3). Model validation and accuracy assessment confirmed that the Ca_ Mg_ K predictors coupled with EBK SVMR model yielded the optimal performance. The R^2^, the root means square error (RMSE) and the mean absolute error (MAE) of the calibrated model Ca_Mg_K- EBK_SVMR model obtained 0.637 (R^2^), 95.479 mg/kg (RMSE) and 77.368 mg/kg (MAE) as against 0.663 (R^2^), 235.974 mg/kg (RMSE) and 166.946 mg/kg (MAE) for Ca_Mg_K-SVMR. Despite that, Ca_Mg_K-SVMR (0.663 mg/kg R^2^) and Ca_Mg-EBK_SVMR (0.643 =  R^2^) obtained a good R^2^ value; their RMSE and MAE results were higher than that of Ca_Mg_K-EBK_SVMR (R^2^ 0.637) (see Table 3). Moreover, the RMSE and MAE of the Ca_Mg-EBK_SVMR (RMSE = 1664.64 and MAE = 1031.49) model are 17.5 and 13.4, bigger than that of Ca_Mg_K-EBK_SVMR. Similarly, the RMSE and MAE of the Ca_Mg-K SVMR (RMSE = 235.974 and MAE = 166.946) model are equally bigger than Ca_Mg_K-EBK_SVMR RMSE and MAE by a margin of 2.5 and 2.2, respectively. The computed RMSE results indicated how concentrated the dataset is from the best fit line. It was observed that the RSME and MAE were higher. According to Kebonye et al.^46^ and john et al.^54^, the closer the RMSE and the MAE are to zero, the better the results. The quantified RSME and MAE values for SVMR and EBK_SVMR were higher. It was observed that consistently, the RSME estimated values were higher than MAE values, suggesting outliers. According to Legates and McCabe^83^, the extent to which the RMSE surpasses the mean absolute error (MAE) is recommended as an indicator of the occurrence of outliers. This implies that the larger the heterogeneity of the dataset, the higher the MAE and RMSE value^38^. The cross-validation accuracy assessment Ca_Mg_K-EBK_SVMR hybrid model predicts Ni content in the urban and peri-urban soil 63.70% accuracy level. This level of accuracy, according to Li et al.^59^ is an acceptable model performance rate. The current results compared to a previous study by Tarasov et al.^36^ whose hybridized model created MLPRK (multi-layer perceptron residual kriging) to the current study EBK_SVMR accuracy assessment indices reported with regards, RMSE (210) and MAE (167.5) were higher than the results we had in the current study (RMSE 95.479, MAE 77.368). However, when the R^2^ (0.637) of the current study is compared to the R^2^ (0.544) of Tarasov et al.^36^, it is clear that the coefficients of determination (R^2^) in this hybrid model is higher. The hybrid model's marginal errors (RMSE and MAE) (EBK SVMR) are two times lower. Similarly, Sergeev et al.^34^ recorded 0.28 (R^2^) for the hybrid model developed (multi-layer perceptron residual kriging), compared to 0.637 (R^2^) for Ni in the current study. The prediction accuracy level of this model (EBK SVMR) is 63.7%, as opposed to 28% obtained by Sergeev et al.^34^. The final map (Fig. 5) created using the EBK _SVMR model and Ca_Mg_K as predictors showed patches of hotspots and a moderate to nickel prediction across the entire study area. This implies that the concentration of Ni in the study area is primarily moderate, with high concentrations in some specific areas.

**Table 3:** Model comparison using diverse prediction models.

Predictors	Models								
	SVMR			EBK _SVMR			EBK_MLR		
	mg/kg								
	R^2^	RMSE	MAE	R^2^	RMSE	MAE	R^2^	RMSE	MAE
Ca	2.78E−01	1478.87	1015.300	6.85E−05	2710.6	1402.38	2.160E−02	6.246	4.425
K	0.306	323.602	228.251	0.079	144.949	120.139	6.000 E−02	6.123	4.324
Mg	0.161	418.771	324.731	0.002	162.293	122.298	1.350 E−01	5.872	4.296
Ca_Mg	0.416	346.868	259.569	0.643	1664.64	1031.49	1.480 E−01	5.829	4.314
Ca_K	0.584	312.264	242.796	0.533	113.175	91.920	6.100 E−02	6.119	4.320
K_Mg	0.511	281.239	196.885	0.523	114.201	90.926	1.350 E−01	5.871	4.302
Ca_Mg_K	0.663	235.974	166.946	**0.637**	**95.479**	**77.368**	1.500 E−01	5.823	4.320

Significant values are in bold.

### Visualization of predicted Nickel via EBK_SVMR model using self-organizing map

**Figure 6:**
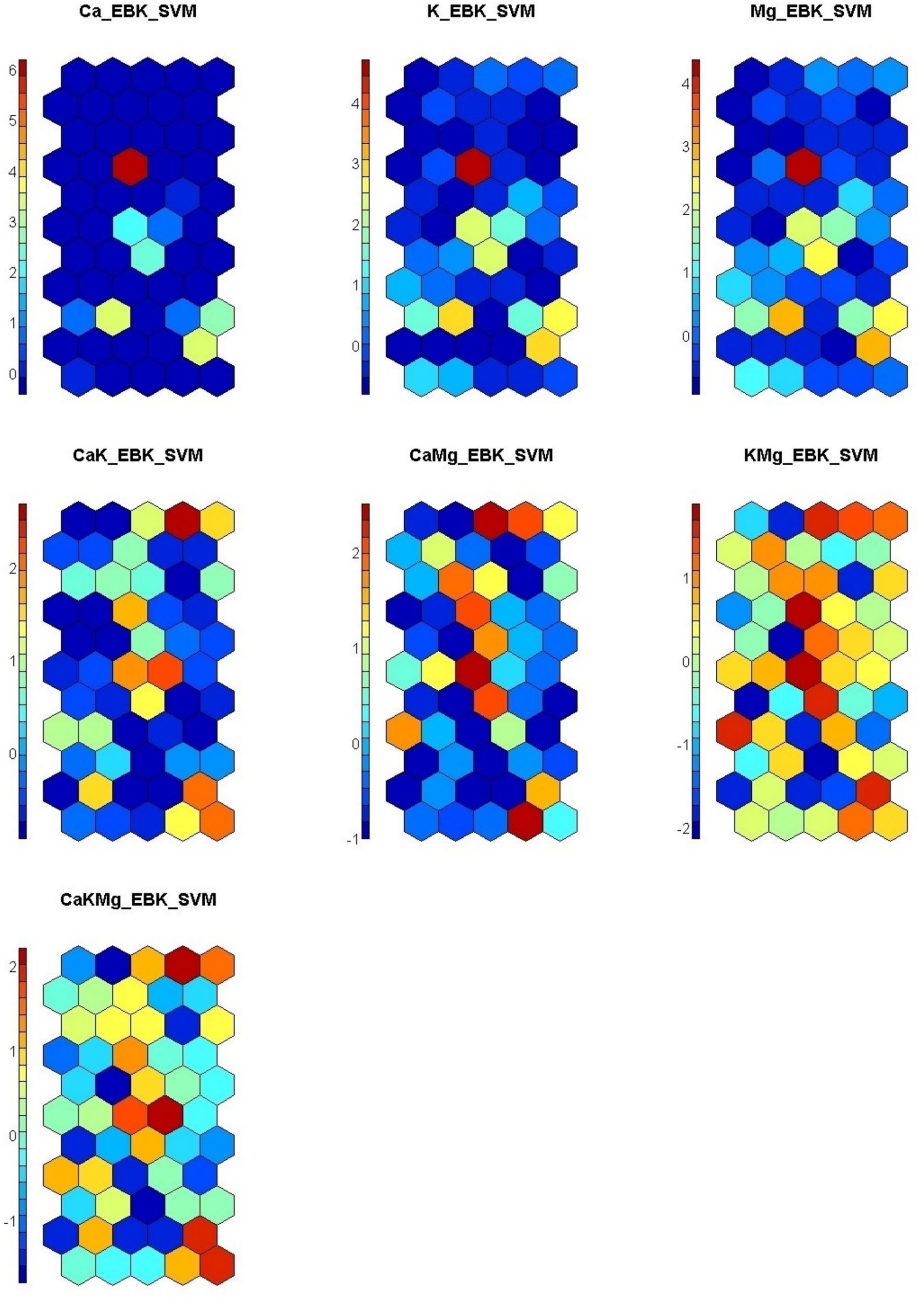
Component planes for each empirical bayesian kriging -support vector machine (EBK_SVM_SeOM) variable output. [The SeOM maps were created with RStudio (Version 1.4.1717: https://www.rstudio.com/).]

**Figure 7:**
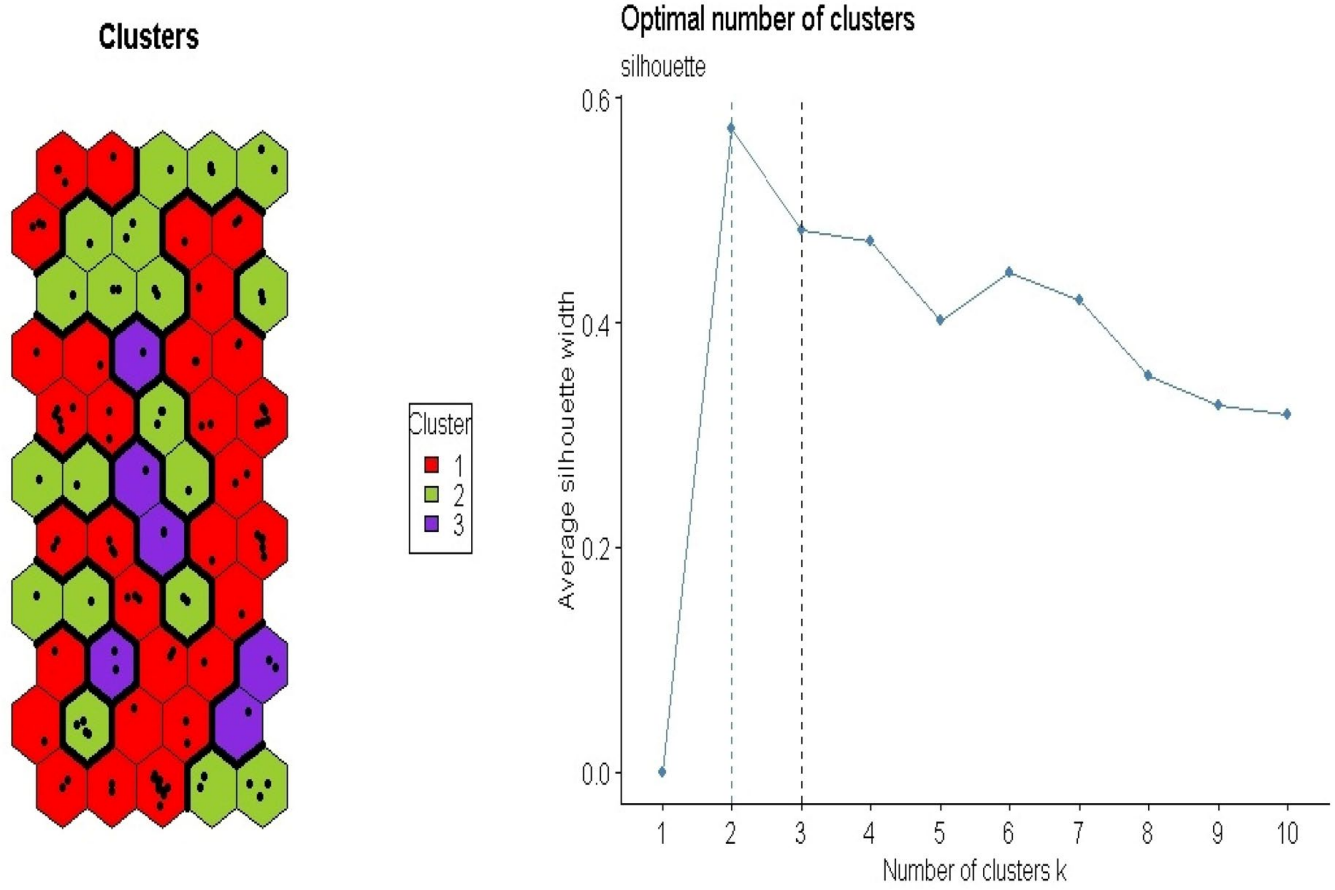
Different clusters classification components [The SeOM map was created with RStudio (Version 1.4.1717: https://www.rstudio.com/).]

Presented in Fig. 6 is the PTEs concentrations as component planes comprising of individual neurons. No component plane exhibited the same colour pattern as shown. However, the appropriate number of neurons per plotted map was 55. The SeOMs were made using various colours, and the more similar the colour pattern, the more comparable the sample attributes are. According to its precise colour scale, the single elements (Ca, K, and Mg) displayed a similar colour pattern with single high neurons and most low neurons. Consequently, CaK and CaMg shared some similarities with very high-level neurons and low to moderate colour patterns. Both models predicted the concentration of Ni in the soil by displaying moderate to high shades of colours such as red, orange, and yellow. The KMg model showed a lot of high colour patterns according to the precise scale and low to moderate patches of colours. The component plane distribution patterns of the models revealed high colour patterns according to the precise color scale ranging from low to high, indicating the potential concentration of Ni in the soil (see Fig. 4). The CakMg model component plane showed a diverse colour pattern from low to high according to the accurate colour scale. In additament, this model's prediction of nickel content (CakMg) is similar to the spatial distribution map of Ni shown in Fig. 5. Both maps revealed high, moderate, and low proportional Nickel concentrations in urban and peri-urban soil. Figure 7 depicts the silhouette method in k-mean groupings on the maps, which are divided into three clusters based on the predicted values in each model. The silhouette method indicated the optimal clustering number. Cluster 1 obtained the most soil samples,74, out of the 115 collected. Cluster 2 received 33 samples, while Cluster 3 received 8 samples. The seven component planes predictor combinations are simplified to allows for proper clustering interpretation. It is difficult to have suitably differentiated cluster patterns in the distributed SeOM map due to the numerous anthropogenic and natural processes that influence soil formation^78^.

## Conclusion

The current research clearly illustrates a modelling technique for nickel concentration in urban and peri-urban soil. The study tested different modelling techniques, combining elements with modelling techniques to obtain the best method for predicting nickel concentration in soil. The SeOM component plane spatial characteristics of the modelling techniques exhibited a high colour pattern spanning between low to high on a precise colour scale, suggesting the concentration of Ni in the soil. However, the spatial distribution map corroborates with the component plane spatial distribution exhibited by EBK_SVMR (see Fig. 5). The results indicated that the support vector machine regression model (Ca Mg K-SVMR) predicted the concentration of Ni in the soil as a unitary model, but validation and accuracy evaluation parameters revealed that the error in terms of RMSE and MAE was very high. The modelling technique employed utilizing EBK_MLR models, on the other hand, was similarly deficient due to the low coefficient of determination (R^2^) values. The use of EBK SVMR and combined elements (CaKMg) resulted in good results with low RMSE and MAE error and a 63.7 percent accuracy level. The results proved that combining the EBK algorithm with a machine learning algorithm can generate a hybrid algorithm that can predict the concentration of PTEs in soil. The results indicated that utilizing Ca Mg K as predictors to predict Ni concentrations in the study area improved Ni prediction in the soil. It implies that the continual application of Ni-based fertilizer and industrial pollution of soil through the steel industry has the tendency to raise the concentration of Ni in the soil. The study revealed the ability of the EBK model to reduce error levels and improve the accuracy of spatial distribution models of soils in urban or peri-urban soil. Generally, we suggest applying the EBK-SVMR model for assessing and predicting PTEs in the soil; moreover, hybridization using EBK with various machine learning algorithms is also recommended. The use of elements as covariates predicted Ni concentration; however, using more covariates will go a long way to improve the model's performance, which can be considered a limitation of the current work. An additional limitation of this study is that number of datasets is 115. As a result, if more data is provided, the performance of the suggested optimized hybridization approaches can be increased.

## References

[1] PlantProbs.net. Nickel in plants and soil https://plantprobs.net/plant/nutrientImbalances/sodium.html (accessed Apr 28, 2021).

[2] Guodong Liu, E. H. Simonne, and Y. L. Nickel Nutrition in Plants | EDIS. *EDis* **2011**.

[3] Liu, G. D. “A New Essential Mineral Element–Nickel.” *Plants Nutr. Fertil. Sci.* **2001**.

[4] Kabata-Pendias, A.; Mukherjee, A. *Trace Elements from Soil to Human*; 2007.

[5] Kasprzak KS (1987). Nickel advances in modern environmental toxicology. Environ. Toxicol..

[6] Cempel M, Nikel G (2006). Nickel: A review of its sources and environmental toxicology. Polish J. Environ. Stud..

[7] Bradl HB (2005). Chapter Sources and origins of heavy metals. Interface Sci. Technol..

[8] Von Burg R (1997). Nickel and some nickel compounds. J. Appl. Toxicol..

[9] Freedman B, Hutchinson TC (1980). Pollutant inputs from the atmosphere and accumulations in soils and vegetation near a nickel–copper smelter at Sudbury, Ontario, Canada. Can. J. Bot..

[10] Manyiwa T, Ultra VU, Rantong G, Opaletswe KA, Gabankitse G, Taupedi SB, Gajaje K (2021). Heavy metals in soil, plants, and associated risk on grazing ruminants in the vicinity of Cu–Ni mine in Selebi-Phikwe, Botswana. Environ. Geochem. Health.

[11] Kabata-Pendias. Kabata-Pendias A. 2011. Trace elements in soils and... - Google Scholar https://scholar.google.com/scholar?hl=en&as_sdt=0%2C5&q=Kabata-Pendias+A.+2011.+Trace+elements+in+soils+and+plants.+4th+ed.+New+York+%28NY%29%3A+CRC+Press&btnG= (accessed Nov 24, 2020).

[12] Almås, A., Singh, B., Agricultural, T. S.-N. J. of & 1995, undefined. The impact of nickel industry in Russia on concentrations of heavy metals in agricultural soils and grass in Soer-Varanger, Norway. *agris.fao.org*.

[13] Nielsen GD (1999). Absorption and retention of nickel from drinking water in relation to food intake and nickel sensitivity. Toxicol. Appl. Pharmacol..

[14] Costa M, Klein CB (1999). Nickel carcinogenesis, mutation, epigenetics, or selection. Environ. Health Perspect..

[15] Agyeman, P. C.; Ahado, S. K.; Borůvka, L.; Biney, J. K. M.; Sarkodie, V. Y. O.; Kebonye, N. M.; Kingsley, J. Trend Analysis of Global Usage of Digital Soil Mapping Models in the Prediction of Potentially Toxic Elements in Soil/Sediments: A Bibliometric Review. *Environmental Geochemistry and Health*. Springer Science and Business Media B.V. 2020. 10.1007/s10653-020-00742-9. 10.1007/s10653-020-00742-9 33094391

[16] Minasny B, McBratney AB (2016). Digital soil mapping: A brief history and some lessons. Geoderma.

[17] McBratney AB, Mendonça Santos ML, Minasny B (2003). On digital soil mapping. Geoderma.

[18] Deutsch.C.V. Geostatistical Reservoir Modeling,... - Google Scholar https://scholar.google.com/scholar?hl=en&as_sdt=0%2C5&q=C.V.+Deutsch%2C+2002%2C+Geostatistical+Reservoir+Modeling%2C+Oxford+University+Press%2C+376+pages.+&btnG= (accessed Apr 28, 2021).

[19] Olea RA (2000). Geostatistics for engineers & earth scientists. Stoch. Environ. Res. Risk Assess..

[20] Gumiaux C, Gapais D, Brun JP (2003). Geostatistics applied to best-fit interpolation of orientation data. Tectonophysics.

[21] Wadoux AMJC, Minasny B, McBratney AB (2020). Machine learning for digital soil mapping: applications, challenges and suggested solutions. Earth-Sci Rev..

[22] Tan K, Wang H, Chen L, Du Q, Du P, Pan C (2020). Estimation of the spatial distribution of heavy metal in agricultural soils using airborne hyperspectral imaging and random forest. J. Hazard. Mater..

[23] Sakizadeh M, Mirzaei R, Ghorbani H (2017). Support vector machine and artificial neural network to model soil pollution: a case study in Semnan Province, Iran. Neural Comput. Appl..

[24] Vega FA, Matías JM, Andrade ML, Reigosa MJ, Covelo EF (2009). Classification and regression trees (CARTs) for modelling the sorption and retention of heavy metals by soil. J. Hazard. Mater..

[25] Sun H, Guo ZX, Guo Y, Yuan YZ, Chai M, Bi RT, Yang J (2017). Prediction of distribution of soil cd concentrations in Guangdong Province, China. Huanjing Kexue/Environmental Sci..

[26] Woodcock CE, Gopal S (2000). Fuzzy set theory and thematic maps: accuracy assessment and area estimation. Int. J. Geogr. Inf. Sci..

[27] Finke PA (2006). Chapter 39 Quality assessment of digital soil maps: producers and users perspectives. Dev. Soil Sci..

[28] Pontius RG, Cheuk ML (2006). A generalized cross-tabulation matrix to compare soft-classified maps at multiple resolutions. Int. J. Geogr. Inf. Sci..

[29] Grunwald S (2009). Multi-criteria characterization of recent digital soil mapping and modeling approaches. Geoderma.

[30] Nelson MA, Bishop TFA, Triantafilis J, Odeh IOA (2011). An error budget for different sources of error in digital soil mapping. Eur. J. Soil Sci..

[31] McBratney AB, Minasny B, ViscarraRossel R (2006). Spectral soil analysis and inference systems: A powerful combination for solving the soil data crisis. Geoderma.

[32] Stumpf F (2017). Uncertainty-guided sampling to improve digital soil maps. CATENA.

[33] Legates DR, McCabe GJ (1999). Evaluating the use of ‘goodness-of-fit’ measures in hydrologic and hydroclimatic model validation. Water Resour. Res..

[34] Sergeev AP, Tarasov DA, Buevich AG, Subbotina IE, Shichkin AV, Sergeeva MV, Lvova OA (2017). High variation subarctic topsoil pollutant concentration prediction using neural network residual kriging. AIP Conf. Proc..

[35] Subbotina IE, Buevich AG, Shichkin AV, Sergeev AP, Tarasov DA, Tyagunov AG, Sergeeva MV, Baglaeva EM (2018). Multilayer perceptron, generalized regression neural network, and hybrid model in predicting the spatial distribution of impurity in the topsoil of urbanized area. AIP Conf. Proc..

[36] Tarasov DA, Buevich AG, Sergeev AP, Shichkin AV (2018). High variation topsoil pollution forecasting in the Russian subarctic: using artificial neural networks combined with residual kriging. Appl. Geochemistry.

[37] Tarasov, D.; Buevich, A.; Shichkin, A.; Subbotina, I.; Tyagunov, A.; Baglaeva, E. Chromium Distribution Forecasting Using Multilayer Perceptron Neural Network and Multilayer Perceptron Residual Kriging. In *AIP Conference Proceedings*; American Institute of Physics Inc., 2018; Vol. 1978, p 440019. 10.1063/1.5044048.

[38] John K (2021). Hybridization of cokriging and gaussian process regression modelling techniques in mapping soil sulphur. CATENA.

[39] Gribov A, Krivoruchko K (2020). Empirical Bayesian Kriging Implementation and Usage. Sci. Total Environ..

[40] Samsonova VP, Blagoveshchenskii YN, Meshalkina YL (2017). Use of empirical Bayesian kriging for revealing heterogeneities in the distribution of organic carbon on agricultural lands. Eurasian Soil Sci..

[41] Fabijańczyk P, Zawadzki J, Magiera T (2017). Magnetometric assessment of soil contamination in problematic area using empirical bayesian and indicator kriging: a case study in upper Silesia, Poland. Geoderma.

[42] John K, Afu SM, Isong IA, Aki EE, Kebonye NM, Ayito EO, Chapman PA, Eyong MO, Penížek V (2021). Mapping soil properties with soil-environmental covariates using geostatistics and multivariate statistics. Int. J. Environ. Sci. Technol..

[43] Li T, Sun G, Yang C, Liang K, Ma S, Huang L (2018). Using self-organizing map for coastal water quality classification: Towards a better understanding of patterns and processes. Sci. Total Environ..

[44] Wang Z, Xiao J, Wang L, Liang T, Guo Q, Guan Y, Rinklebe J (2020). Elucidating the differentiation of soil heavy metals under different land uses with geographically weighted regression and self-organizing map. Environ. Pollut..

[45] Hossain Bhuiyan MA, Chandra Karmaker S, Bodrud-Doza M, Rakib MA, Saha BB (2021). Enrichment, sources and ecological risk mapping of heavy metals in agricultural soils of dhaka district employing SOM PMF and GIS Methods. Chemosphere.

[46] Kebonye NM, Eze PN, John K, Gholizadeh A, Dajčl J, Drábek O, Němeček K, Borůvka L (2021). Self-organizing map artificial neural networks and sequential gaussian simulation technique for mapping potentially toxic element hotspots in polluted mining soils. J. Geochemical Explor..

[47] Weather Spark. Average Weather in Frýdek-Místek, Czechia, Year Round - Weather Spark https://weatherspark.com/y/83671/Average-Weather-in-Frýdek-Místek-Czechia-Year-Round (accessed Sep 14, 2020).

[48] Kozák, J. Soil Atlas of the Czech Republic. **2010**, 150.

[49] Vacek O, Vašát R, Borůvka L (2020). Quantifying the pedodiversity-elevation relations. Geoderma.

[50] Krivoruchko, K. *Empirical Bayesian Kriging*; 2012; Vol. Fall 2012.

[51] Vapnik V (1995). The nature of statistical learning theory. Technometrics.

[52] Li Z, Zhou M, Xu LJ, Lin H, Pu H (2014). Training sparse SVM on the core sets of fitting-planes. Neurocomputing.

[53] Cherkassky, V.; Mulier, F. *Learning from Data: Concepts, Theory, and Methods: Second Edition*; 2006. 10.1002/9780470140529.

[54] John K, Isong IA, Kebonye NM, Ayito EO, Agyeman PC, Afu SM (2020). Using machine learning algorithms to estimate soil organic carbon variability with environmental variables and soil nutrient indicators in an alluvial soil. Land.

[55] Vohland M, Besold J, Hill J, Fründ HC (2011). Comparing different multivariate calibration methods for the determination of soil organic carbon pools with visible to near infrared spectroscopy. Geoderma.

[56] Fraser, S. J.; Dickson, B. L. *A New Method for Data Integration and Integrated Data Interpretation: Self-Organising Maps*; 2007.

[57] Melssen, W. J.; Smits, J. R. M.; Buydens, L. M. C.; Kateman, G. Using Artificial Neural Networks for Solving Chemical Problems Part II. Kohonen Self-Organising Feature Maps and Hopfield Networks. *Chemometrics and Intelligent Laboratory Systems*. Elsevier, Amsterdam, 1, 1994, pp 267–291. 10.1016/0169-7439(93)E0036-4.

[58] Kooistra L, Wanders J, Epema GF, Leuven RSEW, Wehrens R, Buydens LMC (2003). The potential of field spectroscopy for the assessment of sediment properties in river floodplains. Anal. Chim. Acta.

[59] Li L, Lu J, Wang S, Ma Y, Wei Q, Li X, Cong R, Ren T (2016). Methods for estimating leaf nitrogen concentration of winter oilseed rape (*Brassica Napus* L.) using in situ leaf spectroscopy. Ind. Crops Prod..

[60] Różański SŁ, Kwasowski W, Castejón JMP, Hardy A (2018). Heavy metal content and mobility in urban soils of public playgrounds and sport facility areas, Poland. Chemosphere.

[61] Bretzel F, Calderisi M (2006). Metal contamination in urban soils of coastal Tuscany (Italy). Environ. Monit. Assess..

[62] Jim CY (1998). Urban soil characteristics and limitations for landscape planting in hong kong. Landsc. Urban Plan..

[63] Birke, M.; Rauch, U.; Chmieleski, J. Environmental Geochemical Survey of the City of Stassfurt: An Old Mining and Industrial Urban Area in Sachsen-Anhalt, Germany. In *Mapping the Chemical Environment of Urban Areas*; John Wiley and Sons, 2011; pp 269–306. 10.1002/9780470670071.ch18.

[64] Khodadoust AP, Reddy KR, Maturi K (2004). Removal of nickel and phenanthrene from kaolin soil using different extractants. Environ. Eng. Sci..

[65] Jakovljevic, M.; Kostic, N.; Antic-Mladenovic, S. *The Availability of Base Elements (Ca, Mg, Na, K) in Some Important Soil Types in Serbia*; 2003. 10.2298/zmspn0304011j.

[66] Orzechowski, M.; Smolczynski, S. *IN SOILS DEVELOPED FROM THE HOLOCENE DEPOSITS IN NORTH-EASTERN POLAND**; -, 2007; Vol. 15.

[67] Pongrac P, McNicol JW, Lilly A, Thompson JA, Wright G, Hillier S, White PJ (2019). Mineral element composition of cabbage as affected by soil type and phosphorus and zinc fertilisation. Plant Soil.

[68] Kingston, G.; Anink, M. C.; Clift, B. M.; Beattie, R. N. *Potassium Management for Sugarcane on Base Saturated Soils in Northern New South Wales*; 2009; Vol. 31.

[69] Santo, L. T., Nakahata, M. H., & Schell, V. P. Santo LT, Nakahata MH, Ito GP and Schell VP (2000).... - Google Scholar https://scholar.google.com/scholar?hl=en&as_sdt=0%2C5&q=Santo+LT%2C+Nakahata+MH%2C+Ito+GP+and+Schell+VP+%282000%29.+Calcium+and+liming+trials+from+1994+to+1998+at+HC%26S.+Technical+supplement+to+Agronomy+Report+83%2C+Hawaiian+Agricultural+Research+Centre. (accessed May 16, 2021).

[70] Burgos P, Madejón E, Pérez-de-Mora A, Cabrera F (2008). Horizontal and vertical variability of soil properties in a trace element contaminated area. Int. J. Appl. Earth Obs. Geoinf..

[71] Olinic T, Olinic E (2016). The effect of quicklime stabilization on soil properties. Agric. Agric. Sci. Procedia.

[72] Madaras, M.; Lipavský, J. *Interannual Dynamics of Available Potassium in a Long-Term Fertilization Experiment*; 2009; Vol. 55. 10.17221/34/2009-pse.

[73] Madaras M, Koubova M, Lipavský J (2010). Stabilization of available potassium across soil and climatic conditions of the Czech Republic. Arch. Agron. Soil Sci..

[74] Pulkrabová J, Černý J, Száková J, Švarcová A, Gramblička T, Hajšlová J, Balík J, Tlustoš P (2019). Is the long-term application of sewage sludge turning soil into a sink for organic pollutants?: Evidence from field studies in the Czech Republic. J. Soils Sedim..

[75] Asare MO, Horák J, Šmejda L, Janovský M, Hejcman M (2021). A medieval hillfort as an island of extraordinary fertile archaeological dark earth soil in the Czech Republic. Eur. J. Soil Sci..

[76] Zádorová T, Penížek V, Šefrna L, Drábek O, Mihaljevič M, Volf Š, Chuman T (2013). Identification of Neolithic to Modern Erosion-Sedimentation Phases Using Geochemical Approach in a Loess Covered Sub-Catchment of South Moravia Czech Republic. Geoderma.

[77] Tlustoš P, Hejcman M, Kunzová E, Hlisnikovský L, Zámečníková H, Száková J (2018). Nutrient status of soil and winter wheat (*Triticum **Aestivum* L.) in response to long-term farmyard manure application under different climatic and soil physicochemical conditions in the Czech Republic. Arch. Agron. Soil Sci..

[78] Wang Z (2020). Elucidating the differentiation of soil heavy metals under different land uses with geographically weighted regression and self-organizing map. Environ. Pollut..

[79] Yan P, Peng H, Yan L, Lin K (2019). Spatial variability of soil physical properties based on GIS and geo-statistical methods in the red beds of the Nanxiong Basin, China. Polish J. Environ. Stud..

[80] Beguin J, Fuglstad GA, Mansuy N, Paré D (2017). Predicting soil properties in the Canadian boreal forest with limited data: Comparison of spatial and non-spatial statistical approaches. Geoderma.

[81] Adhikary PP, Dash CJ, Bej R, Chandrasekharan H (2011). Indicator and probability kriging methods for delineating Cu, Fe, and Mn contamination in groundwater of Najafgarh Block, Delhi, India. Environ. Monit. Assess..

[82] John K (2021). Mapping soil properties with soil-environmental covariates using geostatistics and multivariate statistics. Int. J. Environ. Sci. Technol..

[83] Eldeiry AA, Garcia LA (2008). Detecting soil salinity in alfalfa fields using spatial modeling and remote sensing. Soil Sci. Soc. Am. J..

